# Axillary Reverse Mapping in Clinically Node-Positive Breast Cancer Patients

**DOI:** 10.3390/cancers15215302

**Published:** 2023-11-06

**Authors:** Masakuni Noguchi, Masafumi Inokuchi, Miki Yokoi-Noguchi, Emi Morioka, Yusuke Haba, Tomoko Takahashi, Akihiro Shioya, Sohsuke Yamada

**Affiliations:** 1 Breast Center, Department of Breast and Endocrine Surgery, Kanazawa Medical University Hospital, Daigaku-1-1, Uchinada, Kahoku 920-0293, Ishikawa, Japan; inokuchi@kanazawa-med.ac.jp (M.I.); miki-y@kanazawa-med.ac.jp (M.Y.-N.); emi-mori@kanazawa-med.ac.jp (E.M.); haba@kanazawa-med.ac.jp (Y.H.); 2 Department of Radiology, Kanazawa Medical University Hospital, Daigaku-1-1, Uchinada, Kahoku 920-0293, Ishikawa, Japan; tomoko-t@kanazawa-med.ac.jp; 3 Department of Clinical Pathology, Kanazawa Medical University Hospital, Uchinada, Kahoku 920-0293, Ishikawa, Japan; a-shioya@kanazawa-med.ac.jp (A.S.); sohsuke@kanazawa-med.ac.jp (S.Y.)

**Keywords:** axillary reverse mapping, axillary lymph node dissection, breast cancer, neoadjuvant chemotherapy

## Abstract

**Simple Summary:**

Axillary reverse mapping (ARM) nodes are involved in a significant proportion of clinically node-positive (cN+) breast cancer patients. However, neoadjuvant chemotherapy (NAC) is effective at decreasing the incidence of nodal metastases in cN+ patients. In the present study, the rates of involvement of ARM nodes in the NAC group were significantly lower than those of the upfront surgery group (36.6% vs. 62.2%, *p* < 0.01). Despite the lower incidence of metastases in ARM nodes in the NAC group, the rate was still too high to warrant the sparing of ARM nodes. On the other hand, ^18^F-FDG-PET/CT was useful to detect a low risk of ARM node metastases after NAC, but it was still not suitable to detect residual metastatic disease of the axilla. Therefore, suspicious ARM nodes must be removed even in the ARM procedure, while it is important to spare ARM lymphatics in order to minimize arm lymphedema.

**Abstract:**

Background: Axillary reverse mapping (ARM) nodes are involved in a significant proportion of clinically node-positive (cN+) breast cancer patients. However, neoadjuvant chemotherapy (NAC) is effective at decreasing the incidence of nodal metastases in cN+ patients. Patients and methods: One hundred forty-five cN+ patients with confirmed nodal involvement on ultrasound-guided fine needle aspiration cytology were enrolled in this study: one group underwent axillary lymph node dissection (ALND) without NAC (upfront surgery group), and the other group underwent ALND following NAC (NAC group). The patients underwent ^18^F-FDG-positron emission tomography/computed tomography (^18^F-FDG-PET/CT) before surgery, as well as an ARM procedure during ALND. Results: the rates of involvement of ARM nodes in the NAC group were significantly lower than those of the upfront surgery group (36.6% vs. 62.2%, *p* < 0.01). Notably, involvement was significantly decreased after NAC in non-luminal-type tumors as compared to the luminal-type (18.4% vs. 48.5%: *p* < 0.01). Moreover, there was a significant difference in ARM node involvement after NAC between patients with or without axillary uptake of ^18^F-FDG (61.5% vs. 32.5%: *p* < 0.01). Conclusions: NAC significantly decreased the risk of ARM node metastases in cN+ patients, but ^18^F-FDG-PET/CT was not suitable to detect residual metastatic disease of the axilla after NAC.

## 1. Introduction

Sentinel lymph node (SLN) biopsy has become a standard procedure for patients with clinically node-negative (cN0) breast cancer. Currently, ALND has been avoided not only in cN0 patients with negative SLN(s) [1,2] but also in selected patients with one or two positive SLNs undergoing either breast-conserving surgery (BCS) with whole-breast irradiation [3] or mastectomy with axillary irradiation [4]. Despite this, ALND cannot be avoided in all patients with invasive breast cancer. ALND is still necessary for maintaining local control in clinically node-positive (cN+) patients with a heavy axillary tumor burden [5].

Axillary reverse mapping (ARM) has been developed as a procedure that delineates and preserves arm-draining nodes and lymphatics during ALND, thereby minimizing arm lymphedema [6,7]. Several randomized studies have demonstrated that the ARM procedure is useful in reducing the occurrence of lymphedema [8,9,10,11,12,13]. However, there have been concerns reported in the literature that the preservation of ARM nodes may result in retained metastasis and inadequate oncological resection in cN+ patients [14].

Nevertheless, neoadjuvant chemotherapy (NAC) leads to a down-staging of axillary lymph node (ALN) status [15]. The risk of metastases in ARM nodes as well as ALNs might be reduced by NAC [16]. On the other hand, ^18^F-FDG-positron emission tomography/computed tomography (^18^F-FDG-PET/CT) has been applied for breast cancer staging. The utilization of ^18^F-FDG-PET/CT for nodal staging in breast cancer has been controversial. However, compared to breast magnetic resonance imaging (MRI), ^18^F-FDG-PET/CT has consistently demonstrated higher accuracy in axillary staging [17,18]. Nevertheless, it has not been used to detect residual metastatic disease in the ARM nodes after NAC. In the present study, we retrospectively evaluated the involvement of ARM nodes in cN+ patients who underwent ALND following NAC in comparison to those who received ALND without NAC. Moreover, ^18^F-FDG-PET/CT was used to detect residual metastatic disease in the ARM nodes after NAC.

## 2. Patients and Methods

### 2.1. Patients

cN+ patients, consisting of patients with palpable suspicious nodes and those with sonographically suspicious nodes, had confirmed nodal involvement on ultrasound-guided fine needle aspiration cytology (FNAC) at the time of enrollment. Patients with distant metastases, inflammatory breast cancer, previous axillary surgery, or an iodine allergy were excluded. Moreover, patients in whom ARM nodes were not detected during ALND were also excluded. In this retrospective study, patients were divided into two groups according to the use of NAC: the first group consisted of cN+ patients who underwent ALND without NAC (upfront surgery group). The second group consisted of cN+ patients who underwent ALND after NAC (NAC group). cN+ patients in either group underwent ALND without SLN biopsy, except for a few patients who underwent NAC after a positive SLN biopsy and then underwent ALND as the second operation. This study was approved by the Ethical Committee of Kanazawa Medical University Hospital. All patients signed informed consent for the surgical procedures, including the ALND and ARM.

### 2.2. Methods

#### 2.2.1. Ultrasonography (US) and US-Guided FNAC

Axillary lymph nodes (ALNs) were sonographically evaluated by using a 13.0–6.0 MHz linear transducer (Hi-vision Prerius; Hitachi Medical Corporation, Tokyo, Japan). The nodal assessment was based on morphology (round, hypoechoic, with loss of central hilum, eccentric cortical hypertrophy) and increased peripheral blood flow. The most suspicious node in the axilla was selected for FNAC. The technique for US-guided FNAC has been previously described [17]. The results of FNAC were classified as positive, negative, or inadequate sampling.

#### 2.2.2. ^18^F-Fluorodeoxyglucose (^18^F-FDG)-Positron Emission Tomography (PET)/Computed Tomography (CT)

The involvement of ALNs and ARM nodes was preoperatively assessed using whole-body ^18^F-FDG-PET/CT. It was performed with a dedicated PET/CT system (Biography Sensation 16: Siemens Medical Solution. Knoxville, TN, USA). The technique has been previously described [17]. Determining ^18^F-FDG uptake was performed through virtual assessment, wherein an abnormal axillary uptake was considered to be a positive uptake.

#### 2.2.3. ALND and ARM Procedure

All of the patients underwent ALND with the fluorescent ARM procedure. In this study, although the ARM procedure is a technique to avoid removing ARM nodes, ALND was systematically performed within the boundaries of a standard ALND, including ARM nodes in both groups. The boundaries of the ALND were defined as the axillary vein superiorly, the lateral border of the subscapularis muscle laterally, and the medial border of the pectoralis minor muscle medially. During ALND, the long thoracic nerve, the thoracodorsal artery, vein, and nerve were preserved, but the lateral thoracic artery and vein were removed. Additionally, the second intercostobrachial nerve was spared as much as possible.

The fluorescent ARM procedure has been described previously in detail [19]. Briefly, 0.1 mL (0.25 mg) of indocyanine green (ICG) was injected subdermally into the inner side of the wrist after induction of general anesthesia. The ARM nodes and lymphatics were identified by using an invisible near-infrared fluorescence imaging system (PhotoDynamic Eye; Hamamatsu Photonics, Hamamatsu, Japan). Although ARM nodes and lymphatics are generally identified by using isosulfan blue, ICG could serve as an alternative to isosulfan blue for ARM procedures [20]. Any fluorescent nodes, including echelon nodes, were considered ARM nodes. The ARM nodes identified within the boundaries of a standard ALND were removed during the dissection.

#### 2.2.4. Systemic Chemotherapy and Hormone Therapy

Treatment using NAC was discussed with the patients for whom chemotherapy had been indicated prior to surgery. The primary regimens for NAC included either fluorouracil, epirubicin, and cyclophosphamide (FEC), followed by docetaxel (DOC), or FEC followed by DOC and Trastuzumab. Adjuvant systemic chemotherapy and hormone therapy were indicated in both the upfront surgery group and the NAC group, based on the molecular type of the tumor.

#### 2.2.5. Histological and Molecular Subtypes of Tumor and Histopathological Examination of ARM Nodes and ALNs

Breast tumors were classified into three main histological subtypes: invasive ductal carcinoma, invasive lobular carcinoma, and a special type. Additionally, tumors were categorized into four molecular subtypes based on their hormone receptor and HER2 status: Luminal-type; Luminal-HER2-type; HER2-type; and Triple-negative-type. In the examination of axillary nodes, ALND specimens, including ARM nodes, were bisected, and one section from each node was subjected to hematoxylin and eosin (H&E) staining. ARM nodes as well as other ALNs containing macrometastases or micrometastases were considered positive, whereas those containing no tumor cells or isolated tumor cells were considered negative.

#### 2.2.6. Statistical Analysis

Chi-square analysis was used to evaluate proportional differences between the groups. Continuous variables were compared between the groups using the Mann–Whitney *U* test. *p* values less than 0.05 were considered significant. All statistical analyses were performed using js-STAR XR+, release 1.4.0 j.

## 3. Results

### 3.1. Patients, Tumors, and Surgical Procedures

Between June 2009 and December 2022, a total of 145 patients with cN+ breast cancer underwent ALND with the ARM procedure. Ten (6.9%) patients were excluded from this study because the ARM nodes were not detected (six patients in the front surgery group and four patients in the NAC group). Of the 145 patients, there were 74 patients in the upfront surgery group and 71 patients in the NAC group. The upfront surgery group received ALND alone, and the NAC group underwent ALND after NAC, except for four patients who underwent NAC after a positive SLN biopsy and then underwent ALND as the second operation. The characteristics of patients, tumors, and surgical procedures are shown in Table 1. The clinical nodal status was significantly advanced in the NAC group in comparison to those in the upfront surgery group. Furthermore, the upfront surgery group had a significantly higher proportion of luminal-type tumors, whereas the NAC group had a significantly higher proportion of HER2-type tumors. The data for the total number of surgical procedures were not statistically different between both groups (Table 1).

### 3.2. Average Numbers of Dissected ALNs and ARM Nodes and Involved ALNs and ARM Nodes

Table 2 shows the average numbers of dissected and involved ALNs and ARM nodes for the upfront surgery group and the NAC group. The average numbers of dissected ALNs and ARM nodes were not statistically different between the two groups. On the other hand, the average numbers of involved ALNs and ARM nodes were significantly higher in the upfront surgery group than the averages for the NAC group. Particularly in the upfront surgery group, 24 (47%) of 51 patients with luminal-type tumors had four or more involved ALNs. Furthermore, the rates of involvement of ALNs and ARM nodes in the NAC group were significantly lower than those of the upfront surgery group (57.7% vs. 100%, *p* < 0.01; 36.6% vs. 62.2%, *p* < 0.01). Despite the lower incidence of metastases in ARM nodes in the NAC group, the rate was still too high to warrant the sparing of ARM nodes. Interestingly, the ratio of involved ARM nodes to dissected ARM nodes was only 16.7% in the upfront surgery group and 9.6% in the NAC group (Table 2).

### 3.3. Involvement of ALNs and ARM Nodes According to Tumor Molecular Subtype in the NAC Group

The effect of NAC was evaluated according to the tumor molecular subtype in the NAC group. This study classified patients into two types: luminal-type and non-luminal-type, including luminal-Her2-type, Her2-type, and triple-negative-type. As shown in Table 3, the rate of involvement of ALN and ARM-node metastases was significantly lower in the non-luminal-type compared with the luminal-type after NAC (36.8% vs. 81.8%: *p* < 0.01; 18.4% vs. 48.5%: *p* < 0.01). ARM nodes were involved only in 7 out of 38 (18.4%) patients with non-luminal-type tumors after NAC. However, of these seven patients, two (28.6%) patients had four or five metastatic ARM nodes.

### 3.4. Involvements of ALNs and ARM Nodes Assessed Using ^18^F-FDG-PET/CT

All of the patients underwent ^18^F-FDG-PET/CT before surgery, except for five patients who underwent enhanced CT in referred hospitals. The association between axillary uptake of ^18^F-FDG and involvement of ALNs and ARM nodes was assessed in the upfront surgery group and the NAC group. In the upfront surgery group, the involvement of ALNs and ARM nodes was not significantly different between patients with positive uptake and those with negative uptake (100% vs. 100%: ns; 67.2% vs. 50%: ns). In contrast, there was a significant difference in ARM node involvement between patients with positive uptake and those with negative uptake in the NAC group (61.5% vs. 32.5%: *p* < 0.01). Thus, ^18^F-FDG-PET/CT was useful to detect a low risk of ARM node metastases after NAC, but it was not suitable to detect residual metastatic disease of the axilla (Table 4).

## 4. Discussion

It has been considered that cN+ patients may not be suitable candidates for preserving ARM nodes due to the risk of involvement. However, it has been reported that NAC leads to a down-staging of axillary status [15], leading to a potential reduction in the risk of metastases in the ARM nodes as well as the ALNs. In fact, a low incidence of metastatic ARM nodes has been reported in cN+ patients treated with NAC in studies using blue dye for identifying ARM nodes [14,16]. Similarly, the present study found that ARM node involvement was significantly decreased by using NAC (62.2% vs. 36.6%: *p* < 0.01). Moreover, NAC was significantly associated with a low risk of ARM node metastases in cN+ patients with non-luminal types of tumors. Interestingly, the proportion, number of involved ARM nodes, and number of dissected ARM nodes were higher in the fluorescent ARM procedure compared to the dye-guided ARM procedure [14]. The fluorescent imaging technique is highly sensitive for the identification of ARM nodes.

NAC has been found to be extremely effective in patients with HER2-positive or triple-negative cancers, achieving nodal pathologic complete response (pCR) in 44–78% of cases [15,21]. In contrast, nodal pCR occurs only in approximately 20% of patients with luminal-type cancer, making it unlikely that NAC alone can avoid the need for ALND in this patient subgroup [22]. The present study found that NAC significantly reduced the risk of metastases in the ALNs and ARM nodes in patients with non-luminal-type cancer in comparison to those with luminal-type cancer. Specifically, in patients with the non-luminal type, NAC reduced the risk of metastases in the ARM nodes to 18.4%, which is lower than the rates observed in the Z0011 trial [3] (27%) and the AMAROS trial [4] (33%). However, it remains unclear whether these patients are appropriate candidates for preserving ARM nodes, as the implications of leaving chemotherapy-resistant tumor cells in the ARM nodes are not yet fully understood [23].

There is a need for imaging techniques to accurately identify axillary nodal metastases. Currently, breast magnetic resonance imaging (MRI) as well as ^18^F-FDG-PET/CT can detect axillary node metastases [17,24]. ^18^F-FDG-PET/CT has consistently demonstrated higher sensitivity, negative predictive value, and accuracy than breast MRI in axillary staging [18]. In the present study, ^8^F-FDG-PET/CT was useful to detect a low risk of ARM node metastases after NAC but was still not suitable to detect residual metastatic disease of the axilla. Therefore, in clinical practice, identified ARM nodes suspicious for malignancy must be removed even in the ARM procedure [25].

**Figure 1:**
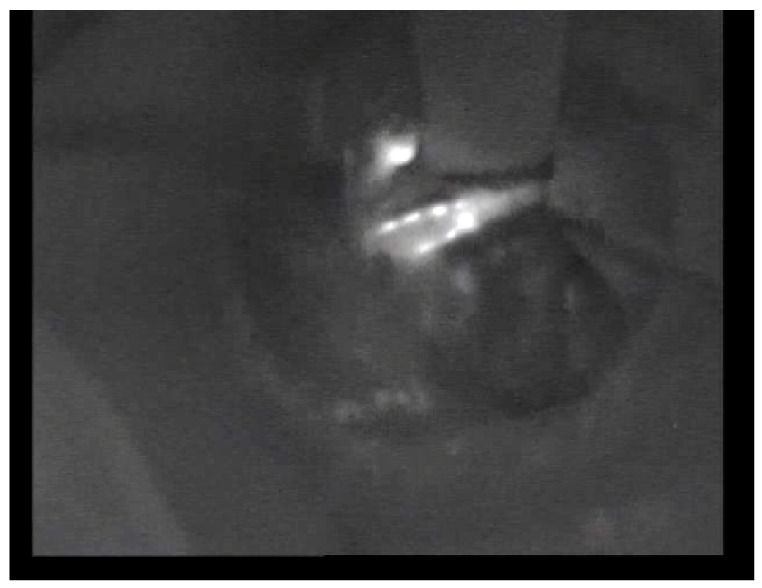
Fluorescent image obtained with infrared camera imaging: fluorescent ARM nodes and efferent lymphatics remain in the axilla after ALND.

Recently, tailored axillary surgery (TAS) has been developed to reduce the axillary tumor volume in cN+ patients after NAC or in the upfront surgical setting [26,27,28]. TAS involves the removal of all palpable, clearly suspicious lymph nodes along with blue/hot SLNs, although imaging-guided localization of clipped nodes is optimal. Thus, it is mandatory to remove palpable suspicious nodes in conservative axillary surgery, even in the era of effective multimodality therapy. Postoperative nodal radiotherapy is effective for obtaining local control in patients with low-volume remaining nodal disease [29]. In order to minimize arm lymphedema, nevertheless, it is important to spare lymphatics draining from the upper extremity during axillary surgery. The fluorescent ARM procedure is highly sensitive to detecting lymphatics from the upper extremity (Figure 1). However, it is not always possible to spare ARM lymphatics during ALND. Casabona et al. [30,31] performed microsurgical lymphatic-venous anastomosis using lymphatic collectors coming from the upper extremity and one of the collateral branches of the axillary vein. In fact, lymphatic microsurgery techniques have been shown to be effective in the treatment of peripheral lymphedema [32]. A limitation of this study is not to assess the incidence of lymphedema after ALND and not to provide survival data. In this study, however, ALND was performed within the boundaries of a standard ALND, including ARM nodes in both groups.

## 5. Conclusions

NAC was significantly associated with a low risk of ARM node metastases in cN+ patients, but it was not enough to spare ARM nodes after NAC. Moreover, ^18^F-FDG-PET/CT was not suitable to detect residual metastatic disease of the axilla after NAC. Therefore, identified ARM nodes suspicious for malignancy must be removed even in the ARM procedure, while ARM lymphatics draining from the upper extremity should be spared in order to minimize arm lymphedema. Further studies are needed to determine whether this modified ARM procedure is oncologically safe and effective in reducing lymphedema in cN+ patients.

## Figures and Tables

**Table 1:** Characteristics of patients, tumors, and surgical procedures.

	Upfront Surgery Group	NAC Group	
	(*n* = 74)	(*n* = 71)	*p* Value
(1) Age (years) (mean ± SD)	64 ± 12	52 ± 10	*p* < 0.01
(2) Menopausal status (pre/post)			
Pre	11 (14.9%)	32 (45.1%)	*p* < 0.01
Post	63 (85.1%)	39 (54.9%)	
(3) Tumor size (cm) (mean ± SD)	3.8 ± 3.7	4.0 ± 7.7	ns
(4) Clinical nodal status			
cN1	60 (81.1%)	46 (64.8%)	*p* < 0.05
cN2	11 (14.9%)	12 (16.9%)	
cN3	3 (4.1%)	13 (18.3%)	
(5) Histological types of tumor			
IDC	63	65	ns
ILC	5	2	
Special type of invasive carcinoma	6	4	
(6) Molecular subtypes of tumor			
Luminal-type	51 (68.9%)	33 (46.5%)	*p* < 0.01
Luminal-HER2-type	9 (12.2%)	13 (18.3%)	
HER2-type	2 (2.7%)	13 (18.3%)	
Triple-negative-type	12 (16.2%)	12 (16.9%)	
(7) Surgical procedures			
Total mastectomy	57 (77.0%)	51 (71.8%)	ns
Partial mastectomy	17 (23.0%)	20 (28.2%)	
SLN biopsy followed by ALND	0	4 ^#^	ns
ALND alone	74	67	

ALND: axillary lymph node dissection; SLN: sentinel lymph node biopsy; NAC: neoadjuvant chemotherapy; IDC: invasive ductal carcinoma; ILC: invasive lobular carcinoma; ns: not significant; ^#^: four patients underwent NAC after positive SLN biopsy.

**Table 2:** Histological involvement of ALNs and ARM nodes.

	Upfront Surgery Group	NAC Group	
	(*n* = 74)	(*n* = 71)	*p*-Value
Axillary lymph nodes #			
(1) No. of dissected ALNs (mean ± SD)	20 ± 8	19 ± 9	ns
(2) No. of involved ALNs (mean ± SD)	5.4 ± 6.7	2.5 ± 4.4	*p* < 0.01
pN0 (0)	0 (0%)	30 (42.3%)	*p* < 0.01
pN1 (1–3)	36 (48.6%)	26 (36.6%)	
pN2 (>3)	38 (51.4%)	15 (21.1%)	
(3) Involvement of ALNs	74 (100%)	41 (57.7%)	*p* < 0.01
ARM nodes			
(1) No. of dissected ARM nodes (mean ± SD)	8.4 ± 5.4	9.4 ± 5.4	ns
(2) No. of involved ARM nodes (mean ± SD)	1.4 ± 2.0	0.9 ± 1.8	*p* < 0.01
pN0 (0)	28 (37.8%)	45 (63.4%)	*p* < 0.01
pN1 (1–3)	41 (55.4%)	19 (26.8%)	
pN2 (>3)	5 (6.8%)	7 (9.9%)	
(3) Involvement of ARM nodes	46 (62.2%)	26 (36.6%)	*p* < 0.01
(4) Ratio involved ARM nodes to/dissected ARM nodes	16.7% (1.4/8.4)	9.6% (0.9/9.4)	

ALNs: axillary lymph nodes; ARM: axillary reverse mapping, NAC: neoadjuvant chemotherapy; ns: not significant; #: Axillary lymph nodes included ARM nodes.

**Table 3:** Histological involvement of ALNs and ARM nodes according to molecular subtype of tumor in NAC group.

Molecular Subtypes	No. of Patients #	Involvement of ALNs	*p* Value	Involvement of ARM Nodes	*p* Value
(1) Luminal-type	33	81.8% (27/33)	*p* < 0.01 48.5% (16/33)		*p* < 0.01
(2) Non-luminal-type * Luminal-Her-2-type	38 13	36.8% (14/38) 4		18.4% (7/38) 2	
Her-2-type	13	5		2	
Triple-negative-type	12	5		3	

ALNs: axillary lymph nodes; ARM: axillary reverse mapping; NAC: neoadjuvant chemotherapy; * non-luminal-type included luminal-Her-2-type, Her-2-type and triple-negative-type; #: five patients did not undergo PET-CT.

**Table 4:** Assessment of involvement in ALNs and ARM nodes using 18F-FDG-PET/CT.

Groups/^18^F-FDG-PET/CT Findings	No. of Patients	Involvement of ALNs	*p* Value	Involvement of ARM Nodes	*p* Value
(1) Upfront surgery group #					
Positive uptake	67	100% (67)	ns	67.2% (45)	ns
Negative uptake	6	100% (6)		50% (3)	
(2) NAC group *					
Positive uptake	26	76.9% (20)	*p* < 0.05	61.5% (16)	*p* < 0.01
Negative uptake	40	47.5% (19)		32.5% (13)	

ALNs: axillary lymph nodes; ARM: axillary reverse mapping; ^18^F-FDG-PET/CT: ^18^F-FDG-positron emission tomography/computed tomography; ns: not significant; #: one patient who did not undergo ^18^F-FDG-PET/CT was excluded; *: five patients who did not undergo ^18^F-FDG-PET/CT were excluded.

## Data Availability

All data generated or analyzed during this study are included in this article. Further inquiries can be directed to the corresponding author.
